# CTGF Triggers Rat Astrocyte Activation and Astrocyte-Mediated Inflammatory Response in Culture Conditions

**DOI:** 10.1007/s10753-019-01029-7

**Published:** 2019-06-10

**Authors:** Ming Lu, Xiao-Feng Yan, Yun Si, Xin-Zhi Chen

**Affiliations:** Department of Neurosurgery, The First People’s Hospital of Xiaoshan District of Hangzhou City, 199 Shixin South Road, Xiaoshan District, Hangzhou, 311200 China

**Keywords:** CTGF, astrocyte, traumatic brain injury, inflammation

## Abstract

To improve clinical outcomes for patients with traumatic brain injury (TBI), it is necessary to explore the mechanism of traumatic brain injury (TBI)–induced neuroinflammation. Connective tissue growth factors (CTGF) have been reported to be involved in the process of inflammatory response or tissue repair, whereas whether and how CTGF participates in the astrocyte-mediated inflammation after TBI remains unclear. In the present study, the TBI-induced activation of astrocytes and augmentation of inflammatory response were simulated by stimulating rat astrocytes with TGF-β1 or CTGF in cultured conditions. TGF-β1 and CTGF both upregulated the expression of GFAP in astrocytes and facilitated the production of inflammatory cytokines and chemokines. Activation of astrocytes by CTGF is in an autocrine manner. According to the results of Boyden chamber assay, CTGF enhanced the recruitment of peripheral blood mononuclear cells (PBMCs) by reactive astrocytes. Besides, CTGF-mediated activation of astrocytes and augmentation of inflammatory response can be terminated by the inhibitor of ASK1 or p38 and JNK. Thus, our data suggested that CTGF could activate astrocytes in an autocrine manner and promote astrocyte-mediated inflammatory response by triggering the ASK1-p38/JNK-NF-κB/AP-1 pathways in astrocytes. Collectively, our study provided evidence that astrocyte-secreted CTGF serves as an amplifier of neuroinflammatory and could be a potential target for alleviating TBI-induced inflammation.

**Electronic supplementary material:**

The online version of this article (10.1007/s10753-019-01029-7) contains supplementary material, which is available to authorized users.

## INTRODUCTION

Traumatic brain injury (TBI) has raised a huge public health concern, affecting individuals around the world [26]. After the initial injury is managed and resolved, 70~80% of TBI patients will suffer from the secondary pathology and develop long-lasting effects (*e.g.*, changes in personality and cognition, anxiety, and depressive-like behaviors) [40]. Moreover, TBI also seems contribute to various chronic degenerative processes (*e.g.*, chronic traumatic encephalopathy, Alzheimer disease, and Parkinson disease) [14]. One of the major driving forces of this secondary pathology appears to be the inflammatory reaction after TBI [13]. By measuring inflammatory biomarkers in serum or cerebrospinal fluid (CSF) of TBI patients, clinicians can predict prognosis and make decisions at the early stage of pathogenesis [15]. Intermediate products and signals of inflammatory response are critical targets for treating TBI [19]. Accordingly, the insights into the inflammatory mechanisms that drive the pathologic and consequent cognitive outcomes help to facilitate clinical outcomes for patients with TBI.

TBI-induced neuroinflammation refers to a cascade reaction encompassing complex interactions of multiple cell types and signal molecules, and astrocytes help orchestrate this response by acting as inflammatory amplifiers [23]. After brain injury, upregulation of glial fibrillary acidic protein (GFAP) induced by mechanical stress would mechanically activate sensitive cation channels and the release of danger signals, including ATP [1], High-mobility group box 1 (HMGB1) [39] and heat shock proteins 60 (HSP60) [41], from damaged cells. Astrocytes could recognize these autocrine or paracrine signals through their cell surface-expressed receptors, transmit these signals to intracellular effectors (NF-κB, p38, JNK, *etc.*) [12], and facilitate the production of inflammatory cytokines (TNF-α [49], IL-6, IL-1β [11], *etc.*) and chemokines (MCP-1 [45], RANTES [31], CXCL1 [51], *etc.*). Subsequently, inflammatory signals would recruit considerable peripheral immune cells across the blood-brain barrier (BBB), leading to pathological features (*e.g.*, edema and diffuse axonal injury). Furthermore, reactive astrocytes could also produce neuroprotective growth factors (*e.g.*, BDNF [21] and IGF-1 [32]), promoting endothelial-mediated neurovascular reconstruction and functional recovery of BBB. There are still questions about how astrocytes get involved in both protective and deleterious neuroinflammations.

Connective tissue growth factors (CTGF, also termed as CCN2), a member of the CCN family, were first discovered in 1991 as a secreted protein in the conditioned media of cultured human umbilical vascular endothelial cells [4]. CTGF have been reported to play various roles (*e.g.*, angiogenesis and mesenchymal growth) in physiological processes [38]. Recently, glial-secreted CTGF has been reported critical for spinal cord repair in a zebrafish model [36]. However, CTGF was over-expressed restrictedly in a small proportion of glial cells, suggesting the delicate orchestration of CTGF function. Indeed, CTGF is abundantly expressed in various tissues, such as skin, blood vessels, liver, and kidney, and its over-expression is often associated with fibrosis, scarring diseases [47], and even tumorigenesis [10]. It has been revealed that CTGF can act as an inflammatory modulator, and it is involved in chronic inflammatory diseases [25]. Liu et al. showed that CTGF activated human synovial fibroblast cells through ASK1, p38/JNK, and NF-κB/AP-1 pathways and upregulated the IL-6 expression in these cells [28]. Since TBI can induce neuroinflammation, activate astrocytes, and cause CTGF accumulation [29], we attempted to explore the connections between CTGF, astrocytes, and inflammatory response in the TBI settings.

In this study, we got preliminary findings that CTGF can activate cultured rat astrocytes and facilitate the production of inflammatory cytokines and chemokines in a dose-dependent manner. CTGF treatment enhanced the recruitment of peripheral blood mononuclear cells (PBMCs) by astrocytes, indicating that CTGF could augment astrocyte-mediated inflammatory response. Like TGF-β1, CTGF activated astrocytes through ASK1-p38/JNK-AP-1/NF-κB signaling pathways. In conclusion, we implicated a role of CTGF in augmenting astrocyte-mediated inflammatory response and provided evidence for considering CTGF as a potential target for alleviating TBI-induced neuroinflammation.

## RESULTS

### CTGF Alone Can Activate Rat Astrocytes in an Autocrine Manner

**Fig. 1:**
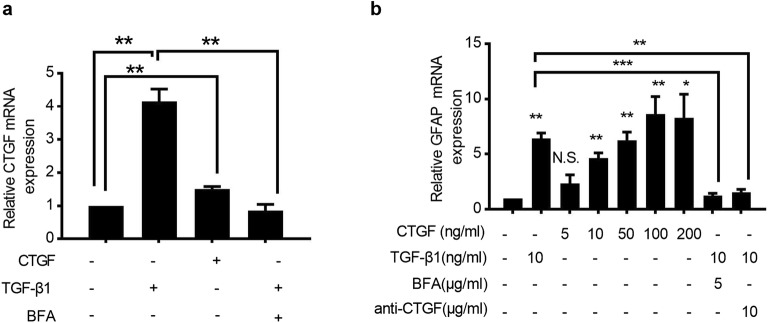
CTGF activated rat astrocytes through an autocrine-dependent manner *in vitro.* RA cells were cultured in the presence of 10 ng/ml recombinant TGF-β1, 5 μg/ml Brefeldin A, 10 μg/ml CTGF neutralizing antibody, or recombinant CTGF with indicated concentrations for 24 h, respectively, and the mRNA expressions of CTGF (**a**) and GFAP (**b**) were examined by quantitative real-time polymerase chain reaction (qRT-PCR). Results are expressed as the mean ± S.E. *p < 0.05, **p < 0.01, and ***p < 0.001. N.S. not significant.

In order to investigate whether CTGF can induce the activation of rat astrocytes, we stimulated rat astrocyte RA with TGF-β1 or CTGF *in vitro*. Consistent with the results described previously [5], TGF-β1 significantly upregulated the transcription of CTGF, indicating that CTGF is a downstream effector of TGF-β1 (Fig. 1a). It has been reported that the expression of TGF-β1 was significantly elevated in CNS after TBI [37], and its over-expression was responsible for the upregulation of GFAP and the activation of astrocytes [54]. Therefore, the mRNA expression of GFAP in RA was measured at 24 h after stimulation with various concentrations of CTGF or 10 ng/ml TGF-β1. We found that CTGF increase the level of GFAP expression in a dose-dependent manner (Fig. 1b).

Interestingly, CTGF alone can increase, even slightly, the auto-production of CTGF by cultured astrocytes (Fig. 1a), probably due to some positive feedback circuit. Upon TGF-β1 induction, CTGF secreted by astrocytes [9] rapidly affected surrounding cells through an autocrine or paracrine manner, resulting in the regulation of cell adhesion and intracellular signaling [48]. To further validate whether auto-production of CTGF activates astrocytes, the mRNA expression of GFAP and CTGF was measured after blocking the secretion of CTGF by Brefeldin A or neutralizing CTGF by the addition of specific antibody in the medium. We found that the activation of CTGF and GFAP by TGF-β1 was remarkably abrogated by Brefeldin A (Fig. 1a, b), and the activation of GFAP by TGF-β1 and CTGF was prevented by CTGF neutralization (Fig. 1b). Collectively, we showed here that CTGF could activate the expression of GFAP in rat astrocytes through an autocrine manner.

### CTGF Alone Can Promote Astrocyte-Mediated Immune Responses

**Fig. 2:**
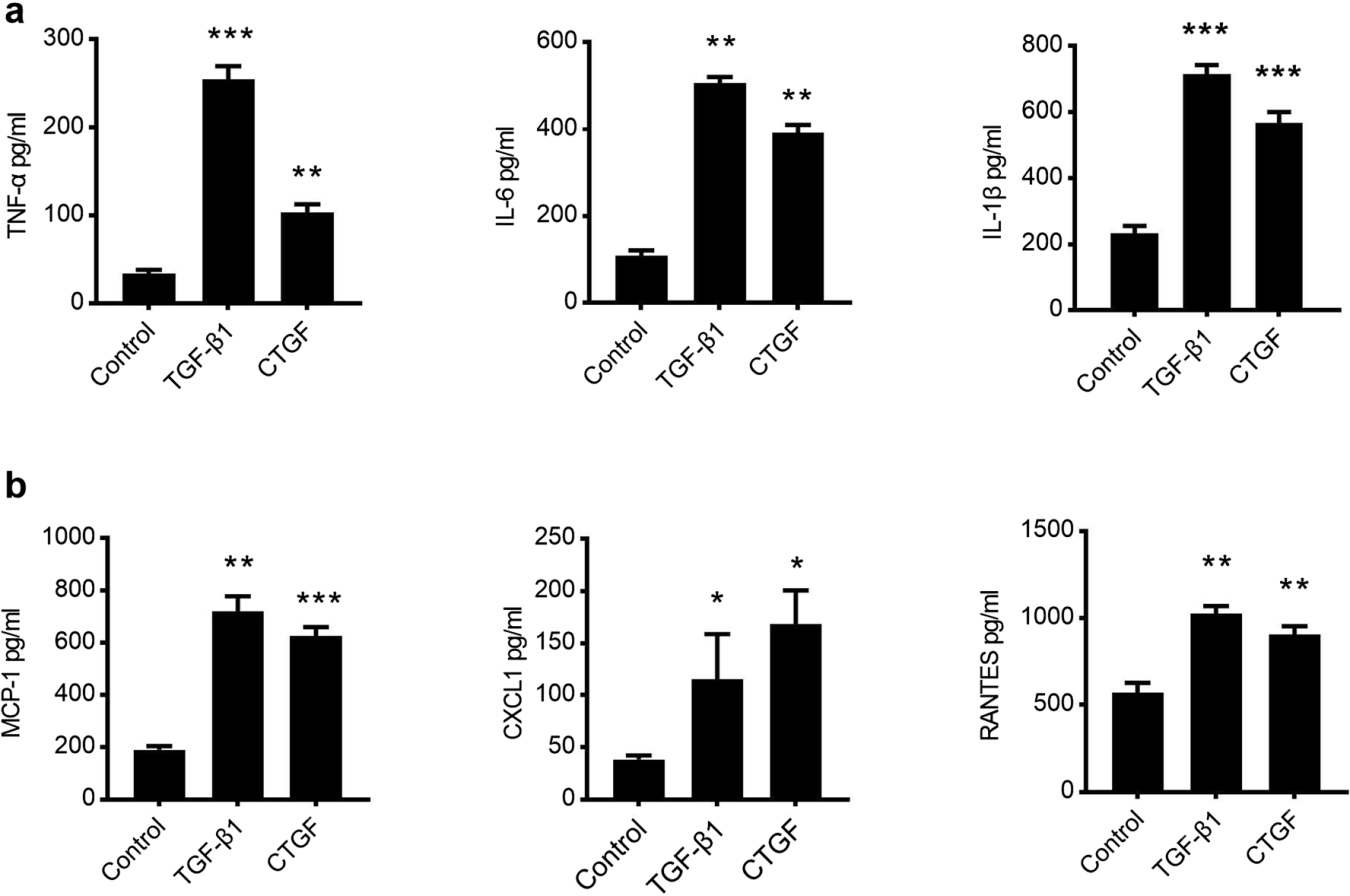
CTGF facilitated the production of cytokines and chemokines by astrocytes. RA cells were cultured in the presence of 10 ng/ml recombinant CTGF or TGF-β1 for 24 h, and supernatants were collected to measure the production of cytokines—TNF-α, IL-6, and IL-1β (**a**), and chemokines—MCP-1, RANTES, and CXCL1 (**b**). Results are expressed as the mean ± S.E. *: *p* < 0.05 as compared with solvent control group. *p < 0.05, **p < 0.01, and ***p < 0.001 as compared with solvent control group.

Upon activation, astrocytes release multiple types of cytokines and chemokines, leading to the initiation of an inflammatory cascade [19]. In order to study the effect of CTGF on the astrocyte-mediated immune response, RA was treated with 10 ng/ml TGF-β1 and 10 ng/ml CTGF. Supernatants were collected 24 h later and assayed for the production of cytokines TNF-α, IL-6, IL-1β, and chemokines CCL2 (MCP-1), CCL5 (RANTES), and CXCL1. Both TGF-β1 and CTGF treatments enhanced the production of TNF-α, IL-6, and IL-1β (Fig. 2a), as well as the production of MCP-1, RANTES, and CXCL1 (Fig. 2b). Unexpectedly, the production of neuroprotective growth factors IGF-1 and BDNF remained unchanged in either TGF-β1 or CTGF groups (Sup Fig. 1), implicating that both TGF-β1 and CTGF stimulations had no effect on astrocyte-mediated brain injury repair.

**Fig. 3:**
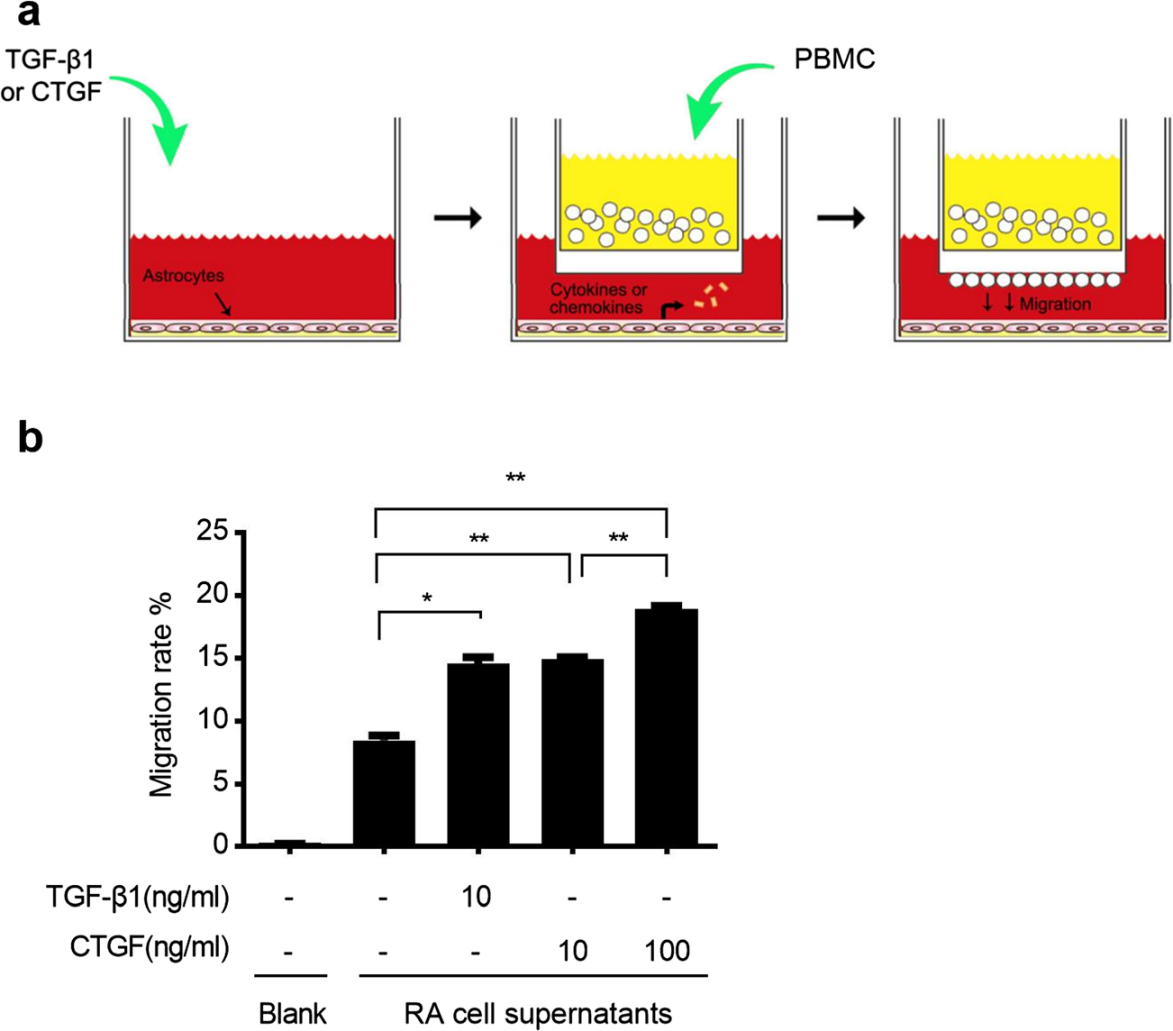
CTGF enhanced the recruitment of PBMCs. **a** Schematic diagram of the Boyden chamber assay. **b** Effects of CTGF treatment on the chemotactic migration of PBMCs cocultured with RA. Number of invaded PBMCs during co-culture with RA was indicated on vertical axis. Results are expressed as the mean ± S.E. *p < 0.05 and **p < 0.01.

The major feature of immune response is the recruitment of immune cells to the damage sites. To confirm whether astrocytes are able to recruit immune cells, we used Boyden chamber techniques to study the migration of peripheral blood mononuclear cells (PBMCs) cocultured with RA (Fig. 3a). After stimulation with TGF-β1 or CTGF, activated RA can enhance the migration of PBMCs (Fig. 3b). Taken together, we found that CTGF facilitated the production of cytokines and chemokines and enhanced the recruitment of PBMCs, leading to locally augmented immune response.

### CTGF-Induced Inflammatory Responses Through ASK1-p38/JNK-NF-κB/AP-1 Pathways

**Fig. 4:**
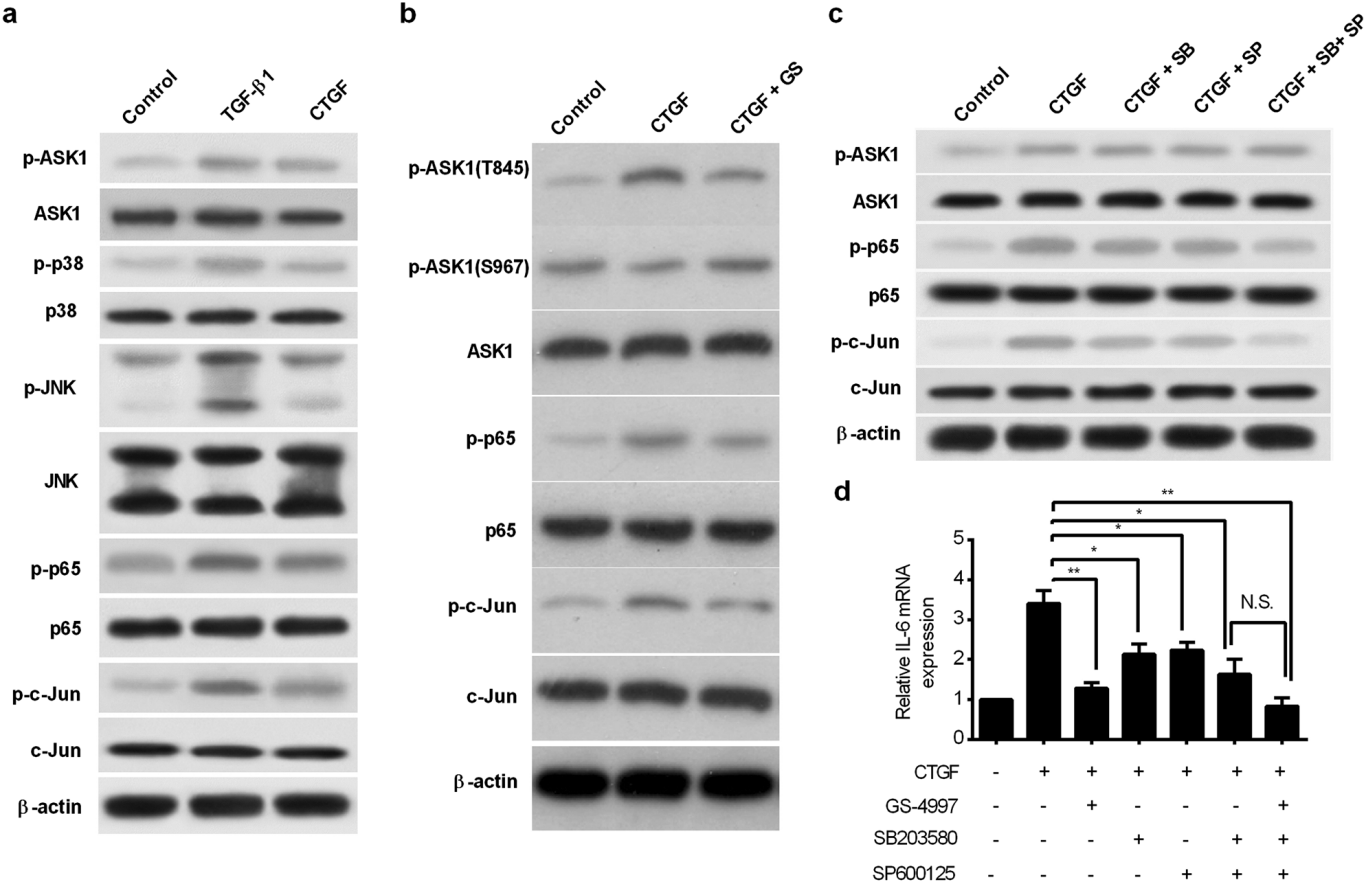
CTGF activated the NF-κB and AP-1 through ASK1-p38/JNK pathways. **a** RA cells were incubated with 10 ng/ml CTGF or TGF-β1 for 24 h, and total protein extracts were collected. The expression of key molecules in ASK, p38/JNK, and NF-κB/AP-1 pathways and their phosphorylation states were examined by Western blotting. **b** RA cells were treated with either 10 μM ASK1 inhibitor GS-4997, or solvent control as indicated, for 30 min prior to stimulation with 20 ng/ml CTGF for 24 h. The expression of ASK, p65, and c-Jun and their phosphorylation states were examined by Western blotting. **c** RA cells were treated with either 5 μM SB20358 or 25 μM SP600125, or DMSO as control, for 30 min prior to stimulation with CTGF for 24 h, and total protein extracts were collected. The expression of ASK, p65, c-Jun and their phosphorylation states were examined by Western blotting. **d** RA cells were treated with chemical inhibitors or solvent control for 30 min prior to stimulation with 10 ng/ml CTGF for 24 h. The mRNA expression of IL-6 was analyzed using qRT-PCR. *p < 0.05 and **p < 0.01 as compared with CTGF group.

In response to extracellular inflammatory signals, apoptosis signal-regulating kinase 1 (ASK1) plays an important role in activating innate immunity [35]. The activation of ASK1 is regulated by phosphorylation at Ther-845, as well as the dephosphorylation at different residues, such as Serine-967 [27]. Here, we found that both TGF-β1 and CTGF treatments increased the phosphorylation at T845 and reduced the phosphorylation at S967, resulting in ASK1 activation (Fig. 4a–b). To investigate the role of ASK1 in the neuroinflammatory response, we used the ASK1-specific inhibitor, GS-4997, to treat astrocytes, and examined the change of astrocyte-mediated immune response in condition of CTGF stimulation. The activation of NF-κB and AP-1 pathways is responsible for the production of cytokines and the amplification of immune response [6, 8]. p65 and c-Jun are key transcriptional factors in the NF-κB and AP-1 pathways, respectively. As indicated by Western blotting, the phosphorylation of p65 at S536 and phosphorylation of c-Jun at T239 were both increased while the expression of the protein p65 and c-Jun did not change after TGF-β1 and CTGF stimulation (Fig. 4a). Notably, the activation of ASK1, p65, and c-Jun induced by CTGF was abrogated by ASK1 inhibition (Fig. 4b), indicating that p65 and c-Jun were both downstream effectors of ASK1. IL-6 is an indicator of NF-κB/AP-1 activation. Quantitative real-time polymerase chain reaction (qRT-PCR) analysis revealed that enhanced expression of IL-6 induced by CTGF was completely impeded by ASK1 inhibition, indicating that ASK1 activation is responsible for astrocyte-mediated NF-κB/AP-1 activation (Fig. 4d).

ASK1 belongs to the MAPKKK family and activates the p38 and JNK pathways *via* MKK3/6 and MKK4/7, respectively [20]. We found activation of JNK (phosphorylated at T183) and p38 (phosphorylated at T180/Y182) after TGF-β1 or CTGF stimulation (Fig. 4a). To further elucidate the role of p38 and JNK in ASK1-mediated immune activation, we blocked the p38 activity using selective inhibitor SB203580 and the JNK activity using SP600125, respectively. We found that addition of either SB203580 or SP600125 alone remarkably inhibited the phosphorylation of p65 and c-Jun induced by CTGF (Fig. 4c). The inhibition was further strengthened when SB203580 and SP600125 were simultaneously added to the medium (Fig. 4c). Notably, neither of these inhibitors can block the phosphorylation of ASK1 (Fig. 4c), indicating that ASK1 acts upstream of p38 and JNK. Likewise, inhibition of p38 by SB203580 or JNK by SP600125 significantly abrogated CTGF-induced IL-6 expression, while simultaneous inhibition of p38 and JNK further strengthened this abrogation (Fig. 4d). Therefore, these results suggested that CTGF can active NF-κB/AP-1 effectors through both ASK1-p38 and ASK1-JNK pathways.

**Fig. 5:**
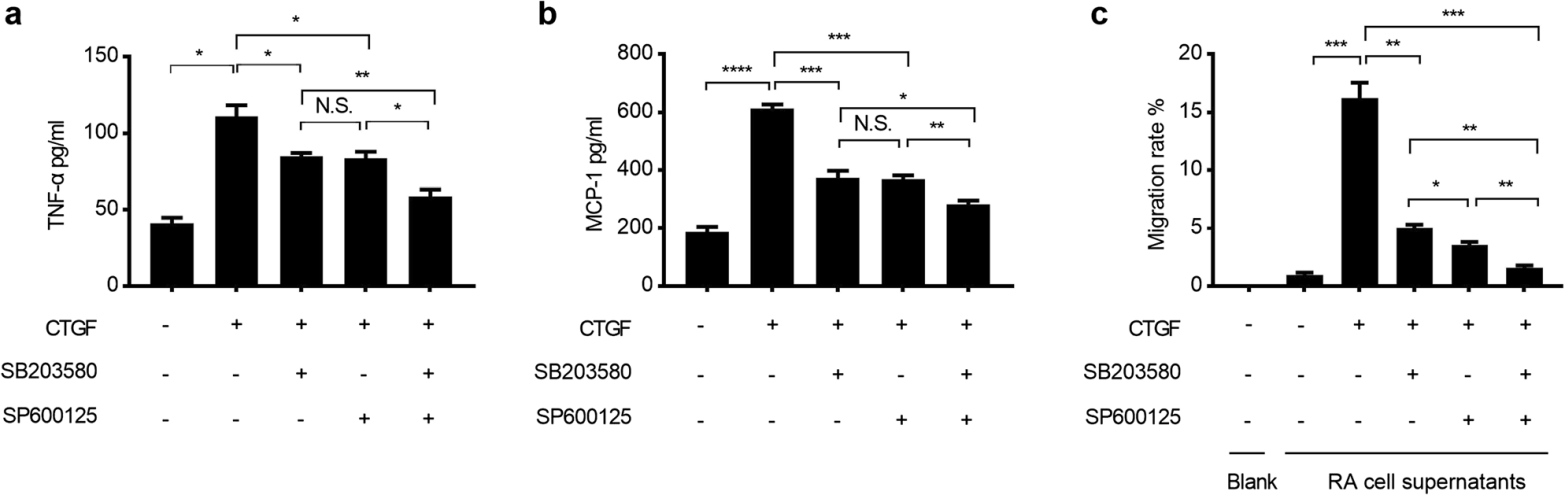
Augmentation of inflammation by CTGF is abrogated by p38 or JNK inhibitors. RA cells were treated with either 5 μM SB20358 or 25 μM SP600125, or DMSO as control, for 30 min prior to stimulation with CTGF. Supernatants were collected to measure the production of TNF-α (**a**) and MCP-1 (**b**), 24 h later. **c** RA cells were seeded in the lower wells of the Boyden chamber, then treated with inhibitors or DMSO for 30 min, followed by the treatment with 10 ng/ml CTGF. Thereafter, the upper wells seeded with PBMCs were placed on top of the lower wells and cells were cocultured for 96 h, then the invaded PBMCs under the membrane were counted. Results are expressed as the mean ± S.E. *p < 0.05, **p < 0.01, and ***p < 0.001 as compared with solvent control group. N.S. not significant.

**Fig. 6:**
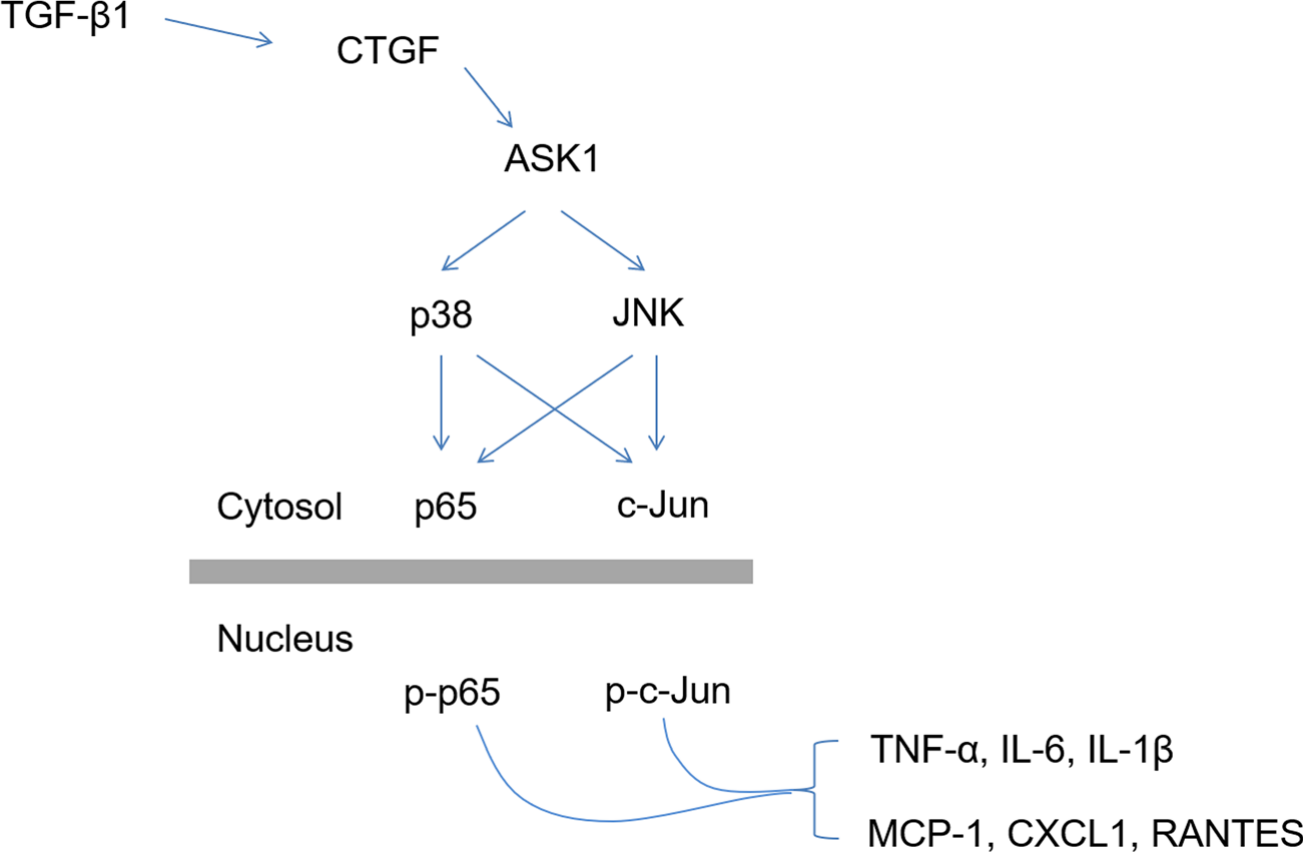
Schematic presentation of the signaling pathways involved in CTGF-induced inflammatory augmentation of astrocytes.

To confirm whether CTGF activates astrocyte-mediated inflammatory response through ASK1-p38/JNK-NF-κB/AP-1 pathways, RA were treated with GS-4997, SB203580, or SP600125 for 30 min and cultured in the presence of CTGF for an extended period of 24 h. Supernatants were collected and assayed for the production of cytokines and chemokines, while migration of cocultured PBMCs was tested in Boyden chamber system. The increase of the production of cytokines—TNF-α (Fig. 5a), IL-6, IL-1β (Sup Fig. 2a), and chemokines—MCP-1 (Fig. 5b), RANTES, and CXCL1 (Sup Fig. 2b) by CTGF was partially abrogated after inhibition of p38 or JNK alone, but completely abrogated after simultaneously inhibition of p38 and JNK. As expected, the inhibition of ASK1 alone almost impeded the production of TNF-α (Sup Fig. 2c). Moreover, enhancement of the migration of cocultured PBMCs was partially impaired in p38 or JNK inhibition group, while completely impaired in p38 and JNK co-inhibition group (Fig. 5c) and in ASK1 inhibition group (Sup Fig. 2d). Taken together, we suggested that CTGF can promote the astrocyte-mediated inflammatory responses through ASK1-p38/JNK-NF-κB/AP-1 pathways (Fig. 6).

## DISCUSSION

Mechanisms regarding how astrocytes get involved in neuroinflammation after brain injury are complex and remain unclear. CTGF emerges as a new target and is intensely studied because of its strong association with chronic inflammatory disorder, such as atherosclerosis, arthritis, inflammatory kidney, and neuroinflammatory diseases [25]. However, the direct contribution of CTGF to astrocyte-mediated TBI-induced neuroinflammation is still unclear. In this study, we performed detailed research of molecular mechanisms and got preliminary data concerning the role of CTGF as an inflammatory amplifier in cultured conditions. We found that auto-production of CTGF can initiate and augment astrocyte-mediated inflammatory response through facilitating the production of inflammatory cytokines and chemokines and enhancing the recruitment of PBMCs. Moreover, our data suggested that CTGF-mediated activation of astrocytes is dependent on ASK1-p38/JNK-AP-1/NF-kB pathways, therefore providing references for manipulating CTGF signaling.

Astrocytes were kind of star-shaped glial cells that widely distributed in the brain and spinal cords. Under physiological conditions, astrocytes are critical in supporting neuronal function, glial transmission, and signaling *via* Ca^2+^ release and uptake, as well as maintaining BBB integrity [23]. In the setting of TBI, an increase in astrocyte reactivity in response to mechanical injury and BBB breakdown is termed astrogliosis [50]. This response involves changes in morphology, increased expression of GFAP, heightened proliferation, and secretion of inflammatory mediators and growth factors. Reactive astrocytes can cause further BBB disruption through augmenting inflammatory response [16], or support repair and regeneration after CNS damage through producing neuroprotective growth factors [33]. Many of these factors act in an autocrine and paracrine fashion to facilitate astrocytic reactivity of the cells surrounding them. Therefore, the effect of astrocytes, either protective or deleterious, were determined by their surrounding microenvironment, illustrated by the variation of localizations, concentrations, and compositions of factors [23]. Still, the functions of these factors involved in this process remained to be determined. TGF-β1 can be synthesized by nearly all cells of the CNS and is upregulated in many CNS disorders including clinical and experimental TBI [37]. It has been reported that TGF-β1 promotes astrogliosis and the transcription of CTGF [17]. Thus, CTGF is an important downstream target of TGF-β1. Moreover, CTGF is also regulated by auto-products [43] and pro-inflammatory mediators, such as TNF-α [22] and IL-1β [34]. All the studies described above implied the critical roles of CTGF in shaping the microenvironment after TBI. In the present study, we found that TGF-β1-induced activation of rat astrocytes was CTGF-dependent. CTGF can upregulate the expression of GFAP and activate astrocytes in an autocrine-dependent manner. Therefore, our results suggested that CTGF could play important roles in amplifying TBI-induced neuroinflammation. However, we do not observe neuroprotective response since there is no significant alteration on the expression of BDNF and IGF-1. Further studies should be performed to examine the alterations of transcriptome to exclude the involvement of astrocytes in brain injury repair. In addition, the comprehensive effect of CTGF on the neuroinflammation and injury repair after TBI should be further examined in animal models.

Inflammation is a cascade response encompassing multiple types of immune cells. Neutrophils are an abundant population of circulating leukocytes that are usually among the first responders to tissue injuries in the periphery and CNS. Neutrophils are rapidly recruited to the CNS after TBI guided by chemokines, such as CXCL1 and CXCL2, and help prepare the damaged environment for repair [44]. CXCR2, the receptor of CXCL1, is dominantly expressed on the surface of neutrophils. Genetic deletion of CXCR2 prevented neutrophils infiltration and attenuated nerve injury shortly after TBI, while it had no effect on the long-term functional recovery of BBB [46]. On the other hand, neutrophils enhance the recruitment of monocytes [24], which are capable of crossing the BBB into the injured brain as a result of MCP-1 and differentiating into macrophages or dendritic cells. MCP-1, another pro-inflammatory chemokines, is significantly increased within 24 h in the CSF of patients with TBI [45]. Examination of MCP-1^−/−^ mice after TBI revealed no changes in lesion size within the first week of injury, but had improved functional recovery after a longer period of 2 to 4 weeks, suggesting a pathogenic role for macrophages during the chronic phase of TBI [45]. T cells play important roles in both innate and adaptive immunities. Although considerable amounts of T cells were recruited to the sites of injury after TBI, their roles are still unclear. RANTES, which is majorly chemotactic for T cells, is elevated in the cortex and the plasma of TBI patients and its concentration may correlate with poor outcome in TBI patients [31]. However, functional studies using RAG1^−/−^ mice, which lack T and B cells, revealed no difference in any pathologic or neurologic parameters [53]. In this study, we found that the productions of CXCL1, MCP-1, and RANTES were all significantly elevated within 24 h after TGF-β1 or CTGF stimulation. Our results implicated that astrocytes activated by CTGF can recruit multiple types of immune cells, including neutrophils, monocytes, and T cells. It might be interesting to further investigate the role of CTGF on specific populations of these immune cells, as well as on the reconstitution of CNS microenvironment, using the animal model of TBI.

In mammalians, ASK1-p38, ASK1-JNK, NF-κB, and AP-1 pathways all contribute to the regulation of innate immunity [2]. ASK1 plays various roles in oxidative stress, endoplasmic reticulum stress, and apoptosis [52]. It has been reported that knockdown of ASK1 prevented the CTGF-mediated activation of p38 and JNK pathways and inhibited the production of IL-6 in human synovial fibroblasts, a major mesenchymal cells contributed to osteoarthritis [28]. Consistent with these results, we found that CTGF-mediated activation of astrocytes is ASK1-dependent. However, deletion of ASK1 blocked only the LPS-induced p38 activation, while had no effect on the activation of JNK and NF-κB signaling [18]. In this study, we found that CTGF-induced activation of ASK1 transduced the signals to both p38 and JNK pathways. Therefore, the difference in signaling transduction might be due to the difference in cell surface receptors and extracellular signals and thus contribute to the divergent functions in astrocytes. Further studies should be performed to examine which receptors are responsible for CTGF-induced activation of astrocytes. In the TBI models, NF-κB and AP-1 pathways regulate cell death and neuroinflammation [42]. NF-κB is a transcriptional factor containing p65 and p50 subunits, while AP-1 is another transcriptional factor containing Jun (c-Jun, JunB, and JunD) and Fos (c-Fos, Fra-1, Fra-2, and FosB) family members. In response to extracellular signals, NF-κB and AP-1 can both be activated by intracellular kinase, such as p38 and JNK, and thus obtain DNA-binding capacity [3, 7]. Inflammatory mediators, such as TNF-α, IL-1β, IL-6, and MCP-1, were potential targets of NF-κB and AP-1. In this study, we found that NF-κB and AP-1 were both activated by CTGF, suggesting that they both play roles in CTGF-mediated activation of astrocytes.

In conclusion, our study indicated a role of CTGF in astrocyte activation and inflammatory augmentation and provided preliminary evidences that CTGF could be valid therapeutic targets for alleviating neuroinflammation and improving the outcomes of TBI.

## MATERIALS AND METHODS

### Cell Lines and Reagents

Rat primary cortical astrocyte cell line RA was purchased from Sigma-Aldrich (Shanghai, China). Recombinant rat CTGF was obtained from R&D Systems with purity > 85% and endotoxin level < 0.1 EU/ml. Recombinant rat TGF-β1 (purity > 90% and endotoxin level < 1 EU/ml) was obtained from Sino Biological, Inc. (Beijing, China). CTGF neutralizing antibody was purchased from R&D Systems. Brefeldin A was purchased from Sigma-Aldrich (Shanghai, China). Selective inhibitors of ASK1 (GS-4997) were purchased from Selleck Inc. (Shanghai, China), and p38 (SB20358) and JNK (SP600125) were purchased from InvivoGen, Inc. (Beijing, China).

### Cell Culture and Stimulation

RA was routinely maintained in DMEM/F12 medium with 10% FBS, penicillin, and streptomycin and cultured at 37 °C in 5% CO_2_. Cells were seeded in 24-well plates with the density of 1 × 10^6^ per well. Prior to stimulation, cells were treated with either 10 μM GS-4997, 5 μM SB20358, 25 μM SP600125, or DMSO as control, for 30 min. Then, supernatants were replaced with refresh DMEM medium containing 10 ng/ml recombinant TGF-β1, 5 μg/ml Brefeldin A, 10 μg/ml CTGF neutralizing antibody, or recombinant CTGF with various concentrations, respectively. Supernatants were collected and assayed for the production of cytokines and chemokines, 24 h later. After stimulation, cells were dissolved in Trizol (Invitrogen, Shanghai, China) for RNA extraction and quantitative real-time polymerase chain reaction (qRT-PCR) analysis. Alternatively, cells were washed with PBS and dissolved in lysis buffer and stored at − 80 °C for Western blotting analysis.

Peripheral blood mononuclear cells (PBMCs) were purified using Ficoll centrifugation (Ficoll-Paque Plus, GE Healthcare) from the blood of adult Sprague-Dawley (SD) rats (obtained from model animal research center, Nanjing University, China). Cells were maintained in RPMI 1640, supplemented with 10% heat-inactivated FBS, penicillin, and streptomycin, and cultured at 37 °C in 5% CO_2_.

### Quantitative Real-time Polymerase Chain Reaction

Total RNA was extracted from RA using a Trizol kit (Invitrogen, Shanghai, China). The reverse transcription reactions were performed using 2 μg of total RNA that was reverse transcribed into cDNA using oligo (dT) and random mixed primers (Tiangen, China). The quantitative real-time PCR (qPCR) analysis was carried out using Taqman® one-step PCR Master Mix (Applied Biosystems, Shanghai, China) as described previously [28]. Sequences for all target gene primers and probes were listed in Table S1 (β-actin was used as internal control). The threshold was set above the non-template control background and within the linear phase of the target gene amplification to calculate the cycle number at which the transcript was detected (denoted CT). Quantification was performed by normalizing Ct values with β-actin Ct and analyzed with the 2^−ΔΔCT^ method [30].

### Western Blotting

Astrocytes were washed once with ice-cold PBS and lysed in RIPA buffer containing a protease inhibitor mixture (Roche Applied Science, China). Protein concentrations were determined using Bradford reagent (Bio-Rad, China). In total, 20 μg of protein extract was resolved by 10–12% SDS-PAGE and proteins were transferred to a polyvinylidene difluoride (PVDF) membrane according to standard protocols. Primary antibodies against p65, p-p65 (S536), c-Jun, p-c-Jun (T239), ASK1, p-ASK 1 (T845), p-ASK1 (S967), p38, p-p38 (T180/Y182), JNK, p-JNK (T183), and β-actin were all purchased from Affinity Bioscience, Inc. (Shanghai, China) and diluted according to the recommended concentration in product manuals. All antibodies were diluted in tris-buffered saline (TBS) containing 0.5% Tween-20 and were incubated overnight at 4 °C. On the following day, the membranes were incubated with horseradish peroxidase-conjugated goat anti-rat IgG (1:5000; Bio-Rad) for 2 h at room temperature. The blots were developed with the use of an ECL detection kit (Invitrogen, Shanghai, China).

### Measurement of Cytokines and Chemokines in Supernatants

Rat IL-1β, IL-6, and TNF-α and MCP-1, RANTES, and CXCL1 in culture supernatants in response to various stimuli were detected by ELISA kits (BD Biosciences) according to the manufacturers’ standard protocols.

### The Boyden Chamber (Chemotaxis) Assay

The Boyden chamber (chemotaxis) assay were performed in a 48-well-modified Boyden chamber (Neuroprobe, Shanghai, China) on 10-μm-thick uncoated Nucleopore membrane (Neuroprobe, Shanghai, China) with a pore diameter of 8 μm. Migration was measured by assessment of the monocyte migration rate through the membrane, where the PBMCs and the astrocytes were grown without direct cell-to-cell contact. About 1 × 10^5^ PBMCs were seeded into the upper wells (inserts) of the chamber, while 1 × 10^6^ astrocytes were seeded into the lower wells. The astrocytes were treated with either inhibitors or DMSO for 30 min, followed by the treatment with 10 ng/ml CTGF. Thereafter, the upper wells were placed on top of the lower wells and cells were cocultured for 96 h, then the invaded PBMCs under the membrane were counted. Controls were performed by omission of the astrocytes. For better clearness, invasion is shown as % of control (= 100%).

### Statistical Analysis

Results were expressed as the mean ± standard deviation (SD). Differences between groups were examined for statistical significance using two-tailed Student’s *t* test. *P* values of less than 0.05 are considered to be significant.

## Electronic supplementary material

ESM 1 (PDF 316 kb)

ESM 2 (PDF 96 kb)
