# Supplementary material for: CTGF Triggers Rat Astrocyte Activation and Astrocyte-Mediated Inflammatory Response in Culture Conditions
Source: Inflammation. 2019 Jun 10;42(5):1693–704. doi: 10.1007/s10753-019-01029-7 (PMC6717176; doi:10.1007/s10753-019-01029-7)

CTGF triggers rat astrocytes activation and astrocyte-mediated inflammatory response in culture conditions

Sup Fig. 1. Effects of CTGF on the secretion of neuroprotective factors. BDNF (a) and IGF-1 (b) in supernatants were assayed by ELISA. N.S., not significant.

Sup Fig. 2. Augmentation of inflammation by CTGF is abrogated by ASK1, p38 or JNK inhibitors. RA cells were treated with 10 μM GS-4997, 5 μM SB20358 or 25 μM as indicated, for 30 minutes, prior to stimulate with CTGF or TGF-β1 for 24 h, and supernatants were collected to measure the production of cytokines—IL-6, IL-1β (a), TNF-α (c) and chemokines—CXCL1, RANTES(b). (d) Effects of ASK1 inhibition on the chemotactic migration of co-cultured PBMCs. *: $p<0.05$, **: $p<0.01$, ***: $p<0.001$, N.S., not significant.

Sup Fig. 1. Effects of CTGF on the secretion of neuroprotective factors.

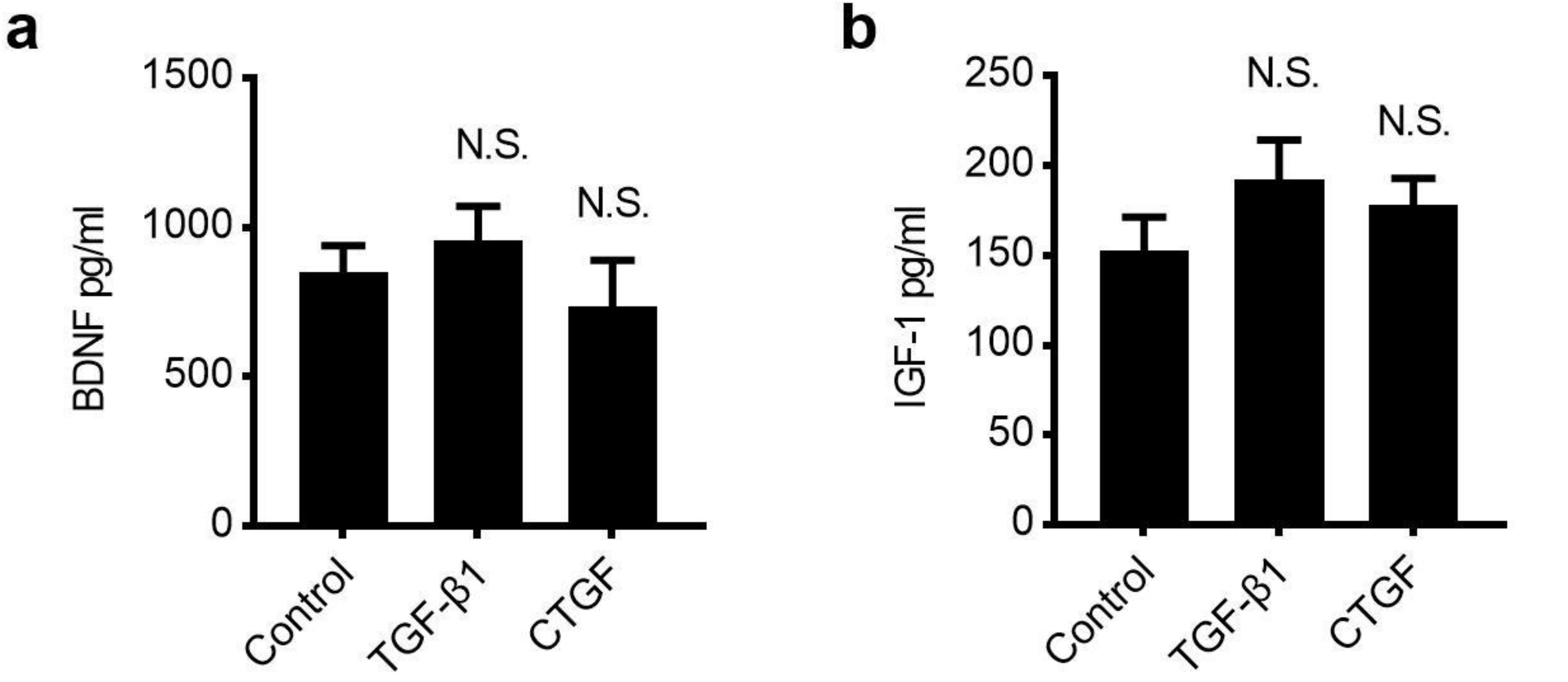

Sup Fig. 2. Augmentation of inflammation by CTGF is abrogated by p38 or JNK inhibitors.

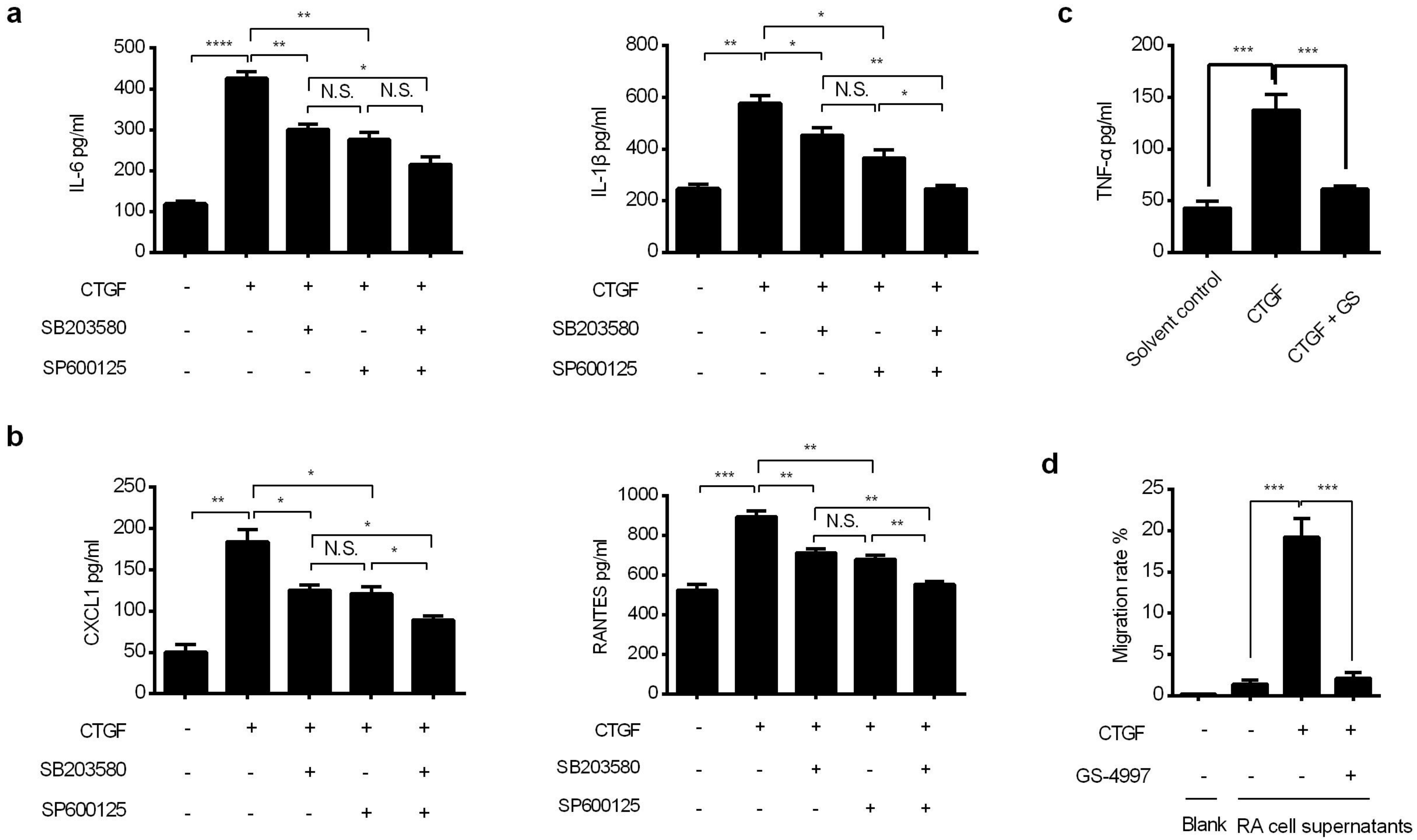

Supplement: Supplementary file 1 — (PDF 316 kb) [file 10753_2019_1029_MOESM1_ESM.pdf]
