# Supplementary material for: CTGF Triggers Rat Astrocyte Activation and Astrocyte-Mediated Inflammatory Response in Culture Conditions
Source: Inflammation. 2019 Jun 10;42(5):1693–704. doi: 10.1007/s10753-019-01029-7 (PMC6717176; doi:10.1007/s10753-019-01029-7)
Supplement: Supplementary file 2 — (PDF 96 kb) [file 10753_2019_1029_MOESM2_ESM.pdf]

Table S1. Primer sequences for qRT-PCR.

Primers	Sequences(5'-3')
Rat CTGF-F	GGAAATGCTGTGAGGAGTGG
Rat CTGF-R	TGGCTCGCATCATAGTTGGG
Rat GFAP-F	GAGAACAACCTGGCTGTGTA
Rat GFAP-R	TCTTGCATGTTACTGGTGGC
Rat IL-6 F	AGCGATGATGCACTGTCAGA
Rat IL-6 R	AGCGATGATGCACTGTCAGA
Rat GAPDH-F	GATGACATCAAGAAGGTGGTGA
Rat GAPDH-R	ACCCTGTTGCTGTAGCCATATTC
